# Inhibition of Diabetes-Related Enzymes by Plant Secondary Metabolites: A Promising Therapeutic Strategy

**DOI:** 10.3390/life16050834

**Published:** 2026-05-19

**Authors:** Oana-Cristina Șeremet, Corina Andrei, Ciprian Pușcașu, Anca Zanfirescu, Georgiana Nițulescu, Cerasela-Elena Gîrd, Octavian-Tudorel Olaru

**Affiliations:** 1 Department of Pharmacology and Clinical Pharmacy, Faculty of Pharmacy, Carol Davila University of Medicine and Pharmacy, 6 Traian Vuia Street, 020945 Bucharest, Romania; oana.seremet@umfcd.ro (O.-C.Ș.); anca.zanfirescu@umfcd.ro (A.Z.); 2 Department of Pharmaceutical Technology and Biopharmacy, Faculty of Pharmacy, Carol Davila University of Medicine and Pharmacy, 6 Traian Vuia Street, 020945 Bucharest, Romania; georgiana.nitulescu@umfcd.ro; 3 Department of Pharmacognosy and Phytotherapy, Faculty of Pharmacy, Carol Davila University of Medicine and Pharmacy, 6 Traian Vuia Street, 020945 Bucharest, Romania; cerasela.gird@umfcd.ro; 4 Department of Pharmaceutical Botany and Cell Biology, Faculty of Pharmacy, Carol Davila University of Medicine and Pharmacy, 6 Traian Vuia Street, 020945 Bucharest, Romania; octavian.olaru@umfcd.ro

**Keywords:** type 2 diabetes mellitus, plant secondary metabolites, α-amylase inhibitors, α-glucosidase inhibitors, PTP1B inhibitors, DPP-4 inhibitors

## Abstract

Diabetes mellitus is a chronic and increasingly prevalent metabolic disorder characterized by persistent hyperglycemia, resulting from defects in insulin secretion, insulin action, or both. Despite the availability of pharmacological agents that effectively manage blood glucose levels, many are associated with adverse effects, limited efficacy over time, and high costs. Consequently, there is growing interest in alternative therapies, especially those derived from traditional medicinal plants, that have long been employed in various cultures for managing diabetes. Recent advances in phytochemistry have identified bioactive plant secondary metabolites with promising antidiabetic properties. This review aims to provide a comprehensive overview of plant-derived compounds that exhibit inhibitory activity against key diabetes-related enzymes, including α-glucosidase, α-amylase, protein tyrosine phosphatase 1B (PTP1B) and dipeptidyl peptidase-4 (DPP-4). These enzymes play critical roles in glucose metabolism and insulin signaling pathways. The review highlights the structural diversity of these natural inhibitors, their mechanisms of action, and their effectiveness in preclinical models. Understanding the molecular interactions and pharmacological profiles of these metabolites may facilitate the development of safer and more effective antidiabetic agents.

## 1. Introduction

Type 2 diabetes mellitus (T2DM) is a chronic and complex metabolic disorder in which persistent hyperglycemia arises from the combined effects of progressive pancreatic β-cell failure and insulin resistance in insulin-responsive tissues such as liver, skeletal muscle and adipose tissue [1]. Clinically, T2DM is diagnosed based on biochemical thresholds. Current American Diabetes Association (ADA) and World Health Organization (WHO) criteria include a fasting plasma glucose over 126 mg/dL, a 2 h plasma glucose over 200 mg/dL after an oral glucose tolerance test, a glycated hemoglobin (HbA1c) level higher than 6.5%, and the absence of autoimmune markers [2,3]. The diagnosis is supported by the presence of hyperglycemic symptoms such as polyuria, polydipsia, polyphagia, and unexplained weight loss, or by hyperglycemic crises such as diabetic ketoacidosis [4]. If left untreated, chronic hyperglycemia can induce a broad spectrum of structural and functional vascular complications through mechanisms involving oxidative stress, advanced glycation end-product (AGE) accumulation, and endothelial dysfunction. These ultimately contribute to the development of retinopathy, nephropathy, neuropathy, and cardiovascular diseases [5,6].

T2DM is a major global health challenge, with its prevalence rising steadily as populations age and sedentary lifestyles and obesity become more common. The growing number of affected individuals results from the combined influence of key metabolic risk factors, including obesity, poor dietary patterns, and reduced physical activity [7]. According to the 2021 International Diabetes Federation (IDF) Diabetes Atlas, the global burden of diabetes is substantial, with an estimated 537 million adults affected worldwide, corresponding to approximately 10.5% of the adult population [8]. These values closely mirror those reported in the U.S. National Diabetes Statistics Report, which indicated that about 38.4 million individuals, or 11.6% of the U.S. population, had diabetes in 2021. Prevalence increased to 14.7% among adults and approached 30% in individuals aged 65 years or older, with more than 8.7 million adults meeting laboratory criteria for diabetes yet remaining undiagnosed [9].

Therapeutic management of T2DM relies on several pharmacological classes, each targeting different aspects of glucose dysregulation. Metformin remains a first-line therapy due to its effectiveness and safety, yet its use can be limited by gastrointestinal intolerance and contraindications such as renal impairment [10,11]. Insulin secretagogues, including sulfonylureas, are inexpensive and widely available, but they carry a risk of hypoglycemia and often promote weight gain [12]. Thiazolidinediones improve insulin sensitivity but may cause fluid retention, weight gain, and cardiovascular concerns [13]. Incretin-based therapies offer better tolerability and weight benefits, though cost and gastrointestinal side effects can restrict their use [14]. Sodium–glucose cotransporter-2 (SGLT2) inhibitors have demonstrated cardiorenal benefits, but they increase the risk of genitourinary infections and require adequate renal function [15]. Maintaining long-term glycemic control remains challenging, as most therapies do not halt the progressive decline in β-cell function and may lose effectiveness over time [10].

Plants and plant-based extracts emerge as a promising alternative—or a complementary support—to standard antidiabetic therapies. Their appeal stems not only from long-standing ethnopharmacological use, but also from practical advantages such as lower cost, wider availability, and generally high patient acceptance [16]. At the same time, the field faces important challenges. Natural products often show considerable variability in their composition depending on the plant species, growing environment, and extraction procedures, which makes standardization difficult. Clinical evidence remains limited, and the pharmacokinetics, safety profiles, and long-term effects of many botanicals are not yet fully characterized. Potential interactions with conventional antidiabetic medications further complicate their clinical use. While plant-derived therapies hold meaningful potential, particularly in resource-limited settings, their integration into evidence-based diabetes care will require rigorous mechanistic studies, well-designed clinical trials, and stronger quality-control frameworks [17,18,19].

This review examines plant-derived compounds and extracts with reported antidiabetic activity, with particular emphasis on their molecular targets and mechanisms of action. We focus on phytochemicals that modulate key pathways involved in glucose homeostasis and discuss how these effects relate to the mechanisms of established antidiabetic drugs. Special attention is given to plant secondary metabolites that inhibit diabetes-related enzymes, including α-amylase, α-glucosidase, PTP1B, and DPP-4. By organizing the available evidence around mechanistic targets, this review aims to clarify the potential therapeutic relevance of these natural compounds and to point out areas where the current evidence remains limited.

## 2. Enzymatic and Molecular Pathways Involved in Glucose Metabolism and Its Dysregulation

Glucose homeostasis is maintained through a complex interplay of enzymatic reactions, hormonal signals, and cellular regulatory pathways. Key enzymes such as α-amylase, α-glucosidase, PTP1B, and DPP-4 play central roles in carbohydrate digestion, insulin signaling, and incretin regulation, which contributes to the regulation of glucose fluctuations after eating. In addition to the core enzymatic pathways, a number of other processes can disturb glucose regulation when they are altered—including oxidative stress, inflammatory pathways, GLUT4 translocation, modulation of insulin secretion by pancreatic β-cells, and the formation of advanced glycation end products.

### 2.1. α-Amylase

Human salivary and pancreatic α-amylases are endo-hydrolases of the glycoside hydrolase family 13 (GH13). They have a three-domain fold. Domain A is a catalytic (β/α)_8 TIM-barrel containing the conserved Asp–Glu–Asp triad and substrate-binding subsites for cleaving internal α-1,4 glycosidic bonds. Domain B is a loop from domain A that shapes the active-site cleft and binds Ca^2+^ for stability. Domain C is a C-terminal β-sandwich implicated in stability and interactions. Human α-amylase is allosterically activated by Cl^−^ and stabilized by Ca^2+^; these ions tune active-site geometry and thermal robustness, features broadly conserved across GH13 subfamilies [20].

α-Amylase initiates starch hydrolysis by endo-cleaving internal α-1,4 linkages of amylose and amylopectin to produce maltose, maltotriose, and α-limit dextrins. Salivary α-amylase begins digestion in the oral phase, while pancreatic α-amylase provides the dominant luminal activity in the small intestine, being the main enzyme for starch digestion; brush-border α-glucosidases then complete hydrolysis to glucose for absorption. Variation in salivary amylase expression/activity (e.g., AMY1 copy number) can modulate postprandial glycemic responses, and recent digestion studies reaffirm a substantial salivary contribution for rapidly digestible starches, with pancreatic amylase remaining the principal enzymatic driver in the intestinal phase [21].

Inhibiting α-amylase markedly slows starch breakdown. For example, complete pharmacologic blockade of pancreatic amylase reduces starch absorption from ~100% to only ~80–85%, so more carbohydrate passes into the distal gut [22]. This leads to blunted postprandial glucose excursions and greater delivery of unabsorbed sugars to the ileum (which also increases incretin release). In practice, α-amylase inhibitors (such as acarbose derivatives) exploit this effect to reduce post-meal glycemia, although excessive blockade can cause carbohydrate malabsorption and gastrointestinal side effects [23].

### 2.2. α-Glucosidase

α-Glucosidases are glycoside hydrolases located in the enterocytes of the small intestine. They catalyze the final step of carbohydrate digestion by hydrolyzing terminal α-1,4 and α-1,6 bonds in oligo- and disaccharides (such as maltose, isomaltose, and sucrose), releasing free glucose for absorption. In effect, α-glucosidases (including maltase, glucoamylase, and sucrase-isomaltase) convert α-amylase digestion products into absorbable monosaccharides. Structurally, they feature a multi-domain architecture with a central (β/α)_8 TIM-barrel catalytic domain containing conserved residues, flanked by auxiliary domains that aid in substrate recognition, stability, and specificity. Many α-glucosidases also rely on metal ions for structural integrity or activity, and their active sites are adapted to accommodate diverse α-glucans [24].

α-Glucosidase inhibitors act competitively, delaying the breakdown of complex carbohydrates and slowing glucose absorption. This results in reduced postprandial blood sugar spikes, as undigested carbohydrates persist longer and undergo delayed hydrolysis—often accompanied by increased distal gut fermentation [25].

### 2.3. Protein Tyrosine Phosphatase 1B (PTP1B)

PTP1B is a non-receptor protein tyrosine phosphatase that acts as a key regulator of insulin and leptin signaling. It dephosphorylates the insulin receptor and insulin receptor substrate after insulin binding. By removing phosphates from tyrosine residues on the activated receptor/substrate complex, PTP1B dampens the downstream signaling cascade. In effect, PTP1B opposes insulin’s action, so higher PTP1B activity leads to weaker insulin signaling [26].

This enzyme adopts a classic α/β fold with a central β-sheet flanked by α-helices and contains the signature HC(X)_5R motif, where the catalytic Cys215 executes nucleophilic attack on phosphotyrosine substrates. A deep active-site pocket confers specificity, while a flexible WPD loop (containing Asp181) closes over the substrate during catalysis. The C-terminal region anchors PTP1B to the endoplasmic reticulum membrane, positioning it to dephosphorylate receptor tyrosine kinases and modulate metabolic signaling [27].

Reducing PTP1B activity enhances insulin signaling and glucose uptake. Genetic deletion or pharmacological inhibition of PTP1B prolongs insulin receptor phosphorylation and promotes the downstream PI3K/Akt pathway. For example, mice lacking PTP1B specifically in skeletal muscle show increased insulin receptor phosphorylation, greater GLUT4-mediated glucose uptake, improved systemic insulin sensitivity and better glucose tolerance [28]. Similarly, global PTP1B-deficient mice have lower body fat and heightened insulin sensitivity [29].

Thus, inhibition of PTP1B counteracts insulin resistance—a strategy explored in drug development to enhance insulin action.

### 2.4. Dipeptidyl Peptidase-4 (DPP-4)

DPP-4 is a widely distributed serine protease that rapidly inactivates the incretin hormones glucagon-like peptide-1 (GLP-1) and glucose-dependent insulinotropic polypeptide (GIP). Incretins are gut-derived peptides that amplify glucose-stimulated insulin secretion and suppress glucagon. DPP-4 cleaves the N-terminus of GLP-1 and GIP shortly after their release, thus limiting their half-lives (GLP-1 is normally <2 min. In this way, DPP-4 ends the insulin-stimulating and glucagon-suppressing actions of incretins, allowing for precise regulation of postprandial glucose levels [30].

DPP-4 is composed of a short cytoplasmic tail, a single transmembrane helix, and a large extracellular domain. The extracellular portion includes an N-terminal eight-bladed β-propeller domain and a C-terminal α/β hydrolase domain that houses the serine-dependent catalytic triad (Ser630, Asp708, His740). DPP-4 forms functional homodimers, with the active site located at the dimer interface, and cleaves N-terminal dipeptides from peptides with a proline or alanine in the penultimate position, including incretins [31].

DPP-4 inhibitors prevent the degradation of GLP-1 and GIP, raising endogenous levels of active incretin hormones. The sustained GLP-1/GIP signaling then enhances glucose-dependent insulin secretion from β-cells and suppresses glucagon secretion, thereby lowering both postprandial and fasting blood glucose [32].

In summary, DPP-4 inhibition extends incretin action and boosts insulin output in response to meals (without causing excess hypoglycemia), which improves glycemic control.

### 2.5. Antioxidant and Anti-Inflammatory Pathways

#### 2.5.1. Oxidative Stress in Diabetes Pathogenesis

Chronic high blood glucose in diabetes leads to overproduction of reactive oxygen species (ROS) (via glucose auto-oxidation, mitochondrial overload, polyol pathway flux, etc.). When ROS generation exceeds the capacity of cellular antioxidants, oxidative stress develops [33]. In diabetes, oxidative stress is a critical mediator of tissue damage. For example, excess ROS interact with lipids and proteins in vascular and pancreatic cells, causing lipid peroxidation and other macromolecular damage. This oxidative damage contributes to β-cell dysfunction, endothelial injury and the development of diabetic complications [34].

Inhibiting ROS-generating enzymes (such as NADPH oxidase) or directly neutralizing ROS with antioxidants (like N-acetylcysteine or superoxide dismutase mimetics) mitigates oxidative damage, preserves insulin signaling, and improve glucose homeostasis. Additionally, reducing ROS can protect endothelial function and delay the progression of diabetic complications, including nephropathy and retinopathy.

Overall, targeting ROS—either at the source or via direct scavenging—represents a promising adjunctive strategy for diabetes management [35,36].

#### 2.5.2. Modulation of Oxidative Pathways

Nuclear Factor Erythroid 2-Related Factor 2 (Nrf2) is a master transcription factor that upregulates the cellular antioxidant response. Under oxidative stress, Nrf2 dissociates from its inhibitor (Keap1) and translocates to the nucleus, where it promotes expression of numerous detoxification and antioxidant enzymes. By inducing genes encoding phase-II enzymes (e.g., glutathione S-transferases, NAD(P)H quinone oxidoreductase) and antioxidant proteins (SOD, catalase, etc.), Nrf2 enhances the cell’s ability to eliminate ROS [37].

Superoxide dismutase (SOD) and catalase (CAT) are key enzymatic defenses against ROS. SOD catalyzes the dismutation of superoxide into hydrogen peroxide, which is further converted by catalase into water and oxygen. These reactions reduce the pool of harmful radicals. In diabetes, activating Nrf2 (to boost SOD/CAT expression) or supplying SOD/CAT mimetics can mitigate oxidative injury and improve cellular resilience against glucose-induced ROS [38].

#### 2.5.3. Anti-Inflammatory Mechanisms

Chronic low-grade inflammation is a hallmark of metabolic syndrome and T2DM. Insulin-responsive tissues (adipose, liver, muscle) and immune cells overproduce cytokines such as tumor necrosis factor alpha (TNF-α) and interleukin (IL)-6. Elevated TNF-α, primarily from adipose tissue, interferes with insulin signaling pathways and induces β-cell apoptosis. Similarly, chronically high IL-6 (from fat and immune cells) is strongly linked to insulin resistance and diabetes progression. These cytokines exacerbate insulin resistance by activating stress kinases and serine phosphorylation of IRS proteins [39].

The transcription factor NF-κB is a central coordinator of inflammatory gene expression. Hyperglycemia and free fatty acids activate NF-κB signaling, which in turn induces TNF-α, IL-6 and other pro-inflammatory mediators. Inhibiting NF-κB (or its upstream kinases) therefore reduces these cytokines. In practice, anti-inflammatory interventions that block NF-κB signaling lower TNF-α/IL-6 levels and improve insulin sensitivity. For example, experimental NF-κB inhibitors or antioxidants attenuate cytokine production and ameliorate inflammation-induced insulin resistance [40].

### 2.6. Other Mechanisms of Glucose Regulation

#### 2.6.1. Enhancement of Glucose Uptake via GLUT4 Translocation

The insulin-stimulated glucose transporter GLUT4 is essential for whole-body glucose disposal. When insulin binds its receptor, the PI3K/Akt signaling cascade is activated; Akt further promotes the translocation of GLUT4-containing vesicles to the plasma membrane of muscle and adipose cells. This increases the number of glucose channels at the cell surface, dramatically enhancing glucose uptake into cells (thus lowering blood glucose). Impaired GLUT4 translocation is a key defect in insulin resistance [41].

AMPK-activating compounds and some plant polyphenols stimulate GLUT4 movement by engaging alternative pathways (such as AMP-activated and small G-protein routes) [42]. Compounds like gingerol and resveratrol enhanced GLUT4 membrane docking in muscle through Rab GTPase–mediated trafficking [43].

Thus, drugs or phytochemicals that activate kinases (e.g., Akt or AMPK) can increase GLUT4 translocation and augment glucose uptake independently of insulin.

#### 2.6.2. Modulation of Insulin Secretion from Pancreatic β-Cells

A major mechanism to boost insulin release is via incretin hormones from the gut. GLP-1, secreted by intestinal L-cells in response to feeding, potently enhances glucose-dependent insulin secretion and also suppresses glucagon and gastric emptying. By binding the GLP-1 receptor on β-cells, GLP-1 amplifies the insulin secretory response to a given glucose load. Interventions that enhance incretin signaling increase β-cell insulin output and improve glycemic control [44].

Other pathways directly stimulate insulin release. For example, sulfonylurea drugs close KATP channels on β-cells, causing membrane depolarization and calcium influx that trigger insulin exocytosis. Nutrients such as glucose itself and amino acids also stimulate insulin secretion via metabolic and ionic effects. In general, agents that depolarize β-cells or mimic nutrient signals enhance insulin release, increasing circulating insulin to facilitate glucose uptake by tissues [45].

#### 2.6.3. Inhibition of Advanced Glycation End Products Formation

AGEs form when chronic hyperglycemia drives non-enzymatic attachment of sugars to proteins, lipids or DNA. This Maillard reaction process creates heterogeneous adducts that accumulate in long-lived tissues. Accumulated AGEs alter protein structure (cross-linking extracellular matrix, modifying collagen, etc.) and bind to receptors (RAGE) on cells, triggering pro-inflammatory and oxidative signaling. As a result, AGEs contribute to the microvascular and macrovascular complications of diabetes (retinopathy, nephropathy, neuropathy, atherosclerosis) by promoting inflammation, endothelial dysfunction and oxidative damage. Thus, AGEs are key mediators of “metabolic memory” and chronic diabetic injury [46].

Preventing AGE formation is a strategy to limit long-term complications. In animal models, pharmacological inhibitors of glycation (such as aminoguanidine) significantly prevented or ameliorated diabetic retinopathy, nephropathy and neuropathy. This demonstrates that blocking AGE accumulation can protect tissues from hyperglycemia-driven damage. Therefore, agents that trap reactive carbonyl intermediates or break AGE crosslinks can attenuate the downstream inflammatory and fibrotic cascades caused by AGEs [47].

## 3. Synthetic and Conventional Antidiabetic Medicines

The history of oral antidiabetic therapy began in the early 1950s when carbutamide, an anti-infective sulfonamide, was found to induce hypoglycemic episodes in treated patients. This unexpected effect led to the recognition of sulfonylureas as agents capable of stimulating insulin secretion. Subsequent structural modifications produced tolbutamide, the first sulfonylurea considered safe and effective for long-term clinical use in T2DM [48]. Several derivatives were subsequently developed and introduced into clinical practice, including glibenclamide, glipizide, gliclazide, and glimepiride, each offering substantially greater hypoglycemic potency and enabling effective glucose control at lower doses. These agents also demonstrated improved binding specificity for the pancreatic β-cell sulfonylurea receptor, resulting in more predictable insulin-secretory responses [49].

The biguanide class followed shortly after the first sulfonylureas, with compounds inspired by galegine, a natural molecule isolated from *Galega officinalis* (goat’s rue), a traditional European medicinal plant. Metformin initially received limited attention because structurally related biguanides such as phenformin and buformin were more potent but were ultimately withdrawn in the late 1970s owing to their high risk of lactic acidosis. Although metformin’s reputation suffered by association, its distinct safety profile, lack of weight gain, low risk of hypoglycemia, and insulin-sensitizing properties gradually gained recognition [50].

The glinides are chemical and functional analogs of sulfonylureas, sharing the same mechanism of stimulating insulin release through closure of β cell K-ATP channels. However, their molecular structure confers a much faster onset and shorter duration of action. Repaglinide, nateglinide, and mitiglinide produce a rapid response rather than sustained stimulation. This makes them particularly useful for targeting postprandial hyperglycemia which reduces the risk of prolonged hypoglycemia compared with sulfonylureas. Their short action profile, however, also means they must be taken with each main meal, and they can still contribute to weight gain, which limits their appeal in some patients [51,52].

Thiazolidinediones (TZDs) or glitazones emerged in the late 1990s as a distinct class of insulin-sensitizing agents acting through activation of the nuclear receptor PPAR-γ. Early representatives such as troglitazone, followed by rosiglitazone and pioglitazone, improved peripheral insulin sensitivity in adipose tissue and skeletal muscle by promoting adipocyte differentiation, enhancing glucose uptake, and reducing circulating free fatty acids. Their ability to target a core defect in T2DM made them an appealing adjunct to metformin or sulfonylureas. However, the class has been limited by safety concerns: troglitazone was withdrawn due to hepatotoxicity, rosiglitazone faced restrictions related to cardiovascular risk signals, and pioglitazone has been associated with weight gain, edema, heart failure risk, and debated bladder cancer concerns [53,54].

Incretin-based therapies comprise two major groups: GLP-1 receptor agonists and DPP-4 inhibitors [55]. GLP-1 receptor agonists such as exenatide, liraglutide, dulaglutide, semaglutide, and lixisenatide mimic the action of native GLP-1, augmenting glucose-dependent insulin secretion, suppressing glucagon release, and slowing gastric emptying to promote satiety. Their main limitations consist of gastrointestinal adverse effects and the need for injectable administration [56]. DPP-4 inhibitors, including sitagliptin, saxagliptin, linagliptin, and alogliptin, prevent enzymatic degradation of endogenous GLP-1 and GIP, providing a more modest but well-tolerated enhancement of incretin function. They are considered weight-neutral and provoke a low risk of hypoglycemia [57].

Several natural products laid the foundation for the development of α-glucosidase inhibitors. *Morus alba* leaves contain potent iminosugars such as 1-deoxynojirimycin (DNJ), a natural α-glucosidase inhibitor long used in traditional medicine to reduce postprandial glucose excursions. In parallel, structurally related pseudooligosaccharides were discovered in *Actinoplanes* species, prompting the development of acarbose [58]. The α-glucosidase inhibitors, including acarbose, miglitol, and voglibose, act locally in the small intestine to reduce carbohydrate digestion and attenuate postprandial hyperglycemia. Although weight neutral and associated with a very low risk of hypoglycemia, their clinical utility is limited by gastrointestinal intolerance and relatively modest effects [59].

The third major class of antidiabetic agents inspired by natural compounds are the SGLT2 inhibitors, also known as gliflozins. Their development began with phlorizin, a natural glucoside of phloretin, which was shown to block renal glucose reabsorption by inhibiting sodium–glucose cotransport. Although phlorizin was unsuitable as a drug due to poor oral bioavailability and non-selective inhibition of SGLT1 and SGLT2, it provided the template for designing selective, orally active SGLT2 inhibitors such as empagliflozin, canagliflozin, dapagliflozin, and ertugliflozin [60]. These drugs lower glucose by promoting glycosuria, with added benefits of weight loss and proven cardiovascular and renal protection. Their use, however, requires awareness of predictable adverse effects, including genital infections, volume depletion, and the rare occurrence of ketoacidosis [61].

Several major antidiabetic drug classes have roots in natural compounds, from the plant-derived precursors of biguanides to the iminosugars that inspired α-glucosidase inhibitors and dihydrochalcones that led to SGLT2 inhibitors. These examples show how natural substances have repeatedly guided the development of effective synthetic therapies.

## 4. Phytochemicals Classified by Mechanistic Target

**Figure 1:**
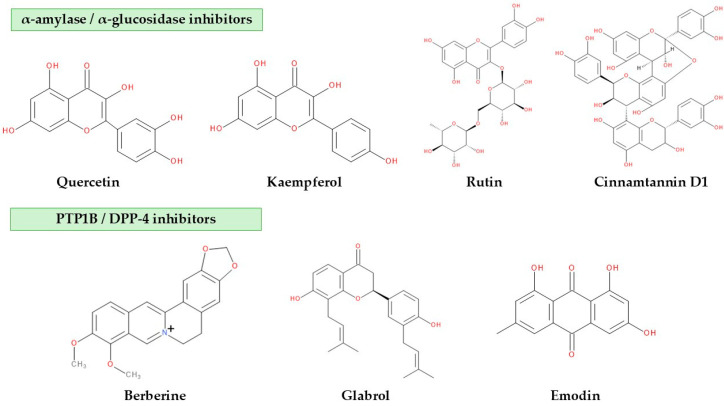
Chemical structures of representative plant secondary metabolites with antidiabetic activity. Legend: PTP1B, protein tyrosine phosphatase 1B; DPP-4, Dipeptidyl peptidase-4.

Plant secondary metabolites encompass a wide range of structurally diverse compounds with the ability to modulate biological pathways relevant to glucose regulation. Because diabetes involves disturbances in several metabolic and signaling mechanisms, grouping phytochemicals according to their molecular targets provides a clearer understanding of how these compounds exert their antidiabetic effects. This chapter focuses on plant-derived metabolites that act on four key enzymes involved in glucose homeostasis—α-amylase, α-glucosidase, PTP1B and DPP-4. The chemical structures of selected key plant secondary metabolites are shown in Figure 1.

**Figure 2:**
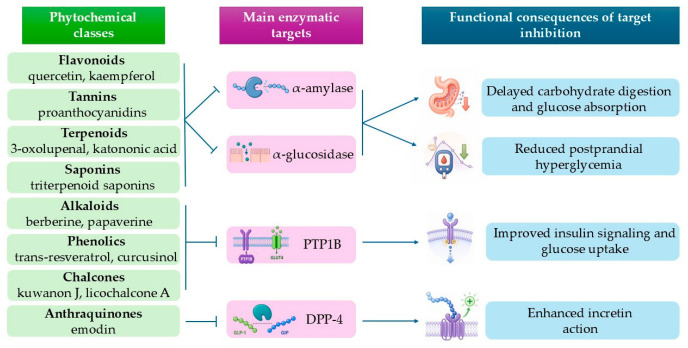
Conceptual summary of phytochemical classes, enzymatic targets, and functional consequences in diabetes. Legend: PTP1B, protein tyrosine phosphatase 1B; DPP-4, Dipeptidyl peptidase-4.

An integrative conceptual overview of the major phytochemical classes, their principal enzymatic targets, and the main functional consequences of target inhibition is presented in Figure 2.

To identify plant secondary metabolites with inhibitory effects on these enzymes, a narrative literature search was surveyed across major scientific databases, including PubMed, Scopus, Web of Science, and Google Scholar. Publications published between 2000 and November 2025 were considered, with emphasis placed on recent studies and those providing mechanistic insights. The search strategy was based on combinations of keywords such as plant secondary metabolites, phytochemicals, diabetes, enzyme inhibition, α-amylase inhibitors, α-glucosidase inhibitors, PTP1B inhibitors, and DPP-4 inhibitors. Studies were selected based on their relevance to the topic, particularly those reporting plant-derived compounds or extracts with inhibitory effects supported by experimental evidence from in vitro, in silico, or in vivo preclinical models. Given the narrative nature of this review, no formal systematic inclusion/exclusion criteria or quantitative quality assessment was applied. Instead, priority was given to studies that provided clear mechanistic data, structure–activity relationship insights, or biologically relevant outcomes.

### 4.1. α-Amylase and α-Glucosidase Inhibitors

Plant-derived inhibitors of α-amylase and α-glucosidase act through well-defined kinetic mechanisms, including competitive, non-competitive, uncompetitive, and mixed-type inhibition. Competitive inhibitors bind directly to the catalytic site, preventing substrate access to key residues involved in glycosidic bond cleavage and thereby reducing substrate turnover. In contrast, non-competitive inhibitors interact with allosteric sites distinct from the catalytic center and decrease catalytic efficiency by inducing conformational changes without directly blocking substrate binding. Uncompetitive inhibitors preferentially bind to the enzyme–substrate complex, stabilizing inactive conformations and reducing overall catalytic activity. Mixed-type inhibitors can interact with both the free enzyme and the enzyme–substrate complex, affecting both substrate binding and catalytic turnover [62,63].

At the molecular level, these inhibitory effects are mediated by non-covalent interactions such as hydrogen bonding, hydrophobic contacts, van der Waals forces, and π–π stacking, which stabilize the enzyme–inhibitor complex and interfere with catalytic function [64]. The specific inhibition pattern is strongly influenced by structural features such as hydroxylation pattern, degree of glycosylation, galloylation, oxidation state, and molecular size, which together determine binding affinity, binding mode, and selectivity toward α-amylase versus α-glucosidase [65].

#### 4.1.1. Flavonoids

Flavonoids constitute the most structurally diverse class of plant secondary metabolites, including flavonols, flavones, flavanones, isoflavones, and anthocyanins [66]. Built on a chromone backbone of two aromatic rings linked by a heterocyclic bridge, they are widely distributed in fruits, vegetables, and medicinal plants. Among them, quercetin and kaempferol are the most abundant flavonols, typically glycosylated at the C-3, C-4, or C-7 positions [66,67,68].

Numerous studies have demonstrated the inhibitory potential of flavonoids against carbohydrate-hydrolyzing enzymes such as α-amylase and α-glucosidase. Li et al. reported that rutin, quercetin, and isoquercetin from *Fagopyrum tataricum* inhibited α-amylase competitively, with isoquercetin showing the strongest affinity [69]. In contrast, Meng et al. [70] and Martínez-González et al. [71] confirmed quercetin as a potent inhibitor, attributing its efficacy to a conjugated C2=C3 double bond, a catechol group on the B-ring, and a planar C-ring. Structural studies revealed that quercetin binding induces minor conformational rearrangements in α-amylase, consistent with a competitive inhibition mechanism [72].

The position and number of hydroxyl substituents greatly influence enzyme–ligand affinity. Kim et al. demonstrated that kaempferol-3-O-[6″-O-(3-hydroxy-3-methylglutaroyl)glucoside] completely inhibited porcine pancreatic α-amylase at 5 mg/mL, while Xiao et al. later confirmed that hydroxylation at 3′ or 3 (flavones) and 6, 3′, or 5′ (flavonols) markedly enhances inhibitory potency [65,73].

Flavonoids also exert significant inhibitory effects on α-glucosidase. Quercetin isolated from *Bruguiera parviflora* exhibited potent inhibition of *Saccharomyces cerevisiae* α-glucosidase (half maximal inhibitory concentration (IC_50_) = 3.4 ± 0.5 μg/mL), alongside anti-inflammatory activity [74]. Comparative analyses of Chinese bayberry (*Morella rubra*) flavonols revealed stronger inhibition by quercetin (IC_50_ = 46.9 μM) compared to kaempferol (IC_50_ = 65.36 ± 0.27 μM), a difference attributed to adjacent hydroxyl groups at C-3′ and C-4′ [75].

In *Sambucus nigra* extracts, quercetin and kaempferol were the most potent inhibitors of both porcine pancreatic α-amylase (IC_50_ = 2.1–3.6 μM) and baker’s yeast α-glucosidase (IC_50_ = 2.6–4.5 μM), surpassing acarbose [76]. Kaempferol inhibited α-glucosidase through a mixed-type mechanism (IC_50_ = 1.16 × 10^−5^ mol L^−1^), forming hydrogen bonds and van der Waals interactions that induce conformational shifts in the enzyme [77]. In *Camellia sinensis*, kaempferol glycosides exhibited dual inhibitory activity: the monoglycoside strongly inhibited α-glucosidase from *Saccharomyces cerevisiae* (IC_50_ = 40.0 μM), whereas the diglycoside potently inhibited hog pancreas α-amylase (IC_50_ = 0.09 μM), highlighting their role in postprandial glucose regulation [78].

Evidence from human intestinal models provides additional insight into the enzyme-specific activity of flavonols. Barber et al. evaluated quercetin, kaempferol, quercetagetin, and galangin using human α-glucosidases (sucrase, maltase, and isomaltase) isolated from differentiated Caco-2/TC7 cells. Among these, quercetagetin showed the strongest inhibition, with activity approaching that of acarbose, followed by galangin and kaempferol, while quercetin was the weakest inhibitor, in contrast to findings from yeast and bacterial enzyme studies. The inhibitory ranking varied depending on the enzyme tested (sucrase > maltase > isomaltase), emphasizing that flavonol activity depends on both structure and enzyme source. These findings demonstrate that data derived from non-human α-glucosidases may overestimate the potency of flavonoid inhibitors, highlighting the importance of using human enzyme systems when assessing antidiabetic potential [79].

Recent evidence provides additional mechanistic insight into quercetin and kaempferol activity. Han et al. screened 29 flavonoids isolated from *Astragali radix* and identified 16 with *Saccharomyces cerevisiae* α-glucosidase inhibitory activity, with quercetin (IC_50_ = 6.65 ± 0.43 μM) and kaempferol (IC_50_ = 38.79 ± 4.96 μM) as the most potent inhibitors [64]. Both compounds acted as mixed-type inhibitors, capable of binding to both the free enzyme and the enzyme–substrate complex. Surface plasmon resonance analysis demonstrated direct enzyme–ligand binding, with quercetin showing higher affinity (Kᴰ = 0.272 μM) than kaempferol (Kᴰ = 356 μM). Molecular docking confirmed interactions with key catalytic residues, including Asp68, Arg212, Asp214, Glu276, and Asp349, through hydrogen bonding and π–π stacking, which contribute to the stabilization of the enzyme–inhibitor complex and suppression of catalytic activity [64]. Inhibitory potency of flavonoids against α-glucosidase is strongly structure-dependent, and enzyme kinetics together with molecular docking show that specific structural features, especially a 3-OH group in the C-ring, a catechol moiety in the B-ring, and overall molecular planarity, promote stronger binding to the catalytic pocket, favor hydrogen bonding with key residues such as Asp68, Asp214, Glu276, and Asp349, and determine whether inhibition follows a competitive, mixed-type, or non-competitive pattern [80].

Taken together, these findings indicate that flavonoid-mediated inhibition of α-amylase and α-glucosidase is closely linked to structure-dependent binding at or near the catalytic pocket, which ultimately governs inhibition mechanism, affinity, and enzyme selectivity.

#### 4.1.2. Tannins

Tannins are astringent, complex polyphenolic compounds broadly classified into condensed tannins (proanthocyanidins), which are composed of flavan-3-ol units, and hydrolysable tannins, which are esters of gallic and/or ellagic acid with a polyol such as glucose [81]. Condensed tannins such as cinnamtannin D1 exhibited remarkable α-glucosidase inhibition (IC_50_ = 0.849 ± 0.014 μM), approximately 53 times more potent than acarbose, acting through a noncompetitive mechanism [82]. Similarly, the hydrolysable tannin 1,2,4-tri-O-galloyl-β-D-glucopyranose, isolated from *Geranium asphodeloides*, demonstrated remarkable α-glucosidase inhibitory activity (IC_50_ = 0.95 ± 0.07 μM), approximately 61-fold stronger than acarbose. Enzyme kinetic analysis revealed an uncompetitive inhibition mechanism, while molecular docking indicated stabilization within the active site through multiple hydrogen bonds formed by the galloyl groups [83]. The similar compound 1,2,3-tri-O-galloyl-β-D-glucopyranose displayed a good activity (IC_50_ = 15.48 ± 0.60 μM), surpassing both acarbose [84]. Among ellagitannins, casuarictin competitively inhibited α-glucosidase (IC_50_ = 2.22 μM) and significantly reduced postprandial blood glucose in vivo [85]. These findings indicate that increasing the degree of polymerization up to trimers and the presence of galloyl groups markedly enhance inhibitory potency.

Tannins play a significant role in the inhibition of α-amylase, and their removal from berry extracts markedly reduces this activity [65]. The inhibitory potency of ellagitannins in tea extracts depends primarily on the position and number of galloyl groups rather than on molecular weight [86]. A synergistic interaction between ellagitannins and anthocyanins has also been observed after berry consumption, enhancing starch degradation inhibition and regulating postprandial glucose levels [87].

Among berry polyphenols, ellagitannins and proanthocyanidins are the most potent α-amylase inhibitors. Variations in enzyme inhibition among different berry extracts are likely due to synergistic interactions between polyphenolic constituents [88]. Proanthocyanidins from *Prunus padus* fruits, composed mainly of (epi)gallocatechin and (epi)catechin subunits and containing both A- and B-type linkages, exhibited potent dual inhibition of porcine pancreas α-amylase and α-glucosidase from rat intestinal acetone powder, with IC_50_ values of 0.19 ± 0.01 μg/mL and 0.18 ± 0.006 μg/mL, respectively, comparable to acarbose [89].

The inhibitory mechanism of tannins against α-glucosidase resembles that of acarbose and voglibose, drugs used clinically to manage non-insulin-dependent diabetes mellitus. Tannic acid and tannin-rich compounds from red wine have been shown to reduce postprandial glucose levels following starch-rich meals [66].

An ellagitannin-rich extract from pomegranate (*Punica granatum*) exhibited strong α-glucosidase (from rat intestinal acetone powder) inhibition and weaker porcine pancreatic α-amylase activity. The main active compounds, punicalagin, punicalin, and ellagic acid, showed IC_50_ values of 140.2, 191.4, and 380.9 μM, respectively. Kinetic analyses indicated a mixed-type inhibition consistent with direct enzyme binding. In a simulated gastrointestinal digestion model, the extract maintained inhibitory activity despite partial ellagitannin degradation [90].

Tannic acid showed potent *Saccharomyces cerevisiae* α-glucosidase inhibition (IC_50_ = 0.35 ± 0.02 μM) through a reversible, mixed-competitive mechanism. Spectroscopic analyses demonstrated direct binding, reduced enzyme surface hydrophobicity, and conformational changes that impaired activity. Molecular docking confirmed hydrogen bonding, electrostatic, and hydrophobic interactions with α-glucosidase [91]. Similarly, other studies have reported that tannic acid exhibits a mixed competitive–noncompetitive inhibition pattern against α-glucosidase, forming stable enzyme–inhibitor complexes primarily stabilized by hydrophobic and electrostatic interactions [92].

Urolithin A, a gut-derived ellagitannin metabolite, showed reversible α-glucosidase inhibition (IC_50_ = 28.03 ± 0.59 μM) through an uncompetitive mechanism. Co-administration with acarbose produced an additive effect, suggesting that urolithin A and related metabolites contribute to the antihyperglycemic activity of ellagitannin-rich diets [93].

Overall, tannin-mediated inhibition of α-amylase and α-glucosidase is primarily associated with their ability to interact with both catalytic and non-catalytic regions of the enzymes. Tannins frequently exhibit non-competitive, uncompetitive, or mixed-type inhibition, reflecting their capacity to bind to the free enzyme as well as the enzyme–substrate complex. Their inhibitory potency is largely influenced by structural features such as the degree of polymerization, the number and position of galloyl groups, and the presence of multiple hydroxyl moieties, which facilitate extensive hydrogen bonding, hydrophobic interactions, and ligand-induced conformational changes, ultimately leading to reduced catalytic efficiency and modulation of postprandial glucose levels [82,83,86,89,90,91].

#### 4.1.3. Terpenoids

Terpenoids represent a structurally diverse class of natural products derived from isoprene units, including monoterpenes, sesquiterpenes, diterpenes, and triterpenes. Monoterpenes (C_10_) are composed of two isoprene units, whereas sesquiterpenes (C_15_), diterpenes (C_20_), triterpenes (C_30_), and tetraterpenes (C_40_) result from the sequential addition of isoprene units to geranyl pyrophosphate [94].

*Nuxia oppositifolia*, traditionally used for diabetes management in Arabian folk medicine, was investigated for its α-amylase and α-glucosidase inhibitory activity. From its n-hexane fraction, two terpenoids, 3-oxolupenal and katononic acid, were isolated. Both showed dual inhibitory effects on starch-hydrolyzing enzymes, with 3-oxolupenal being more potent (IC_50_ = 46.2 μg/mL for α-amylase and 62.3 μg/mL for α-glucosidase) than katononic acid (IC_50_ = 52.4 μg/mL and 88.6 μg/mL, respectively). Fluorescence quenching and molecular docking confirmed strong enzyme binding through hydrogen bonding and hydrophobic interactions [95].

In *Rhododendron minutiflorum*, five new triterpenoids, two rare omphalane-type sesquiterpenoids, and twenty-five known compounds were isolated from leaves and stems. Most triterpenoids exhibited notable α-glucosidase inhibition (IC_50_ = 6.97–229.3 μM), while structure–activity-relationship analysis indicated that oxidation at C-3, C-8, or C-11–C-13 in the ursane-type skeleton enhanced inhibitory potency [96].

In *Cirsium setosum*, bioassay-guided fractionation of the petroleum ether extract led to the isolation of eight taraxastane-type triterpenoids, including three new compounds. Among them, 3β-hydroxy-30-hydroperoxy-20-taraxastene, 3β-hydroxy-22α-methoxy-20-taraxastene, 3β,22-dihydroxy-20-taraxastene, and 3β-hydroxy-20-taraxasten-22-one exhibited the strongest α-glucosidase inhibitory activity, with IC_50_ values ranging from 17.49 ± 1.42 μM to 26.98 ± 0.89 μM, compared to 42.52 ± 0.32 μM for acarbose. The study highlighted the taraxastane skeleton as a key structural motif conferring enhanced inhibitory potency [97].

In *Paramignya trimera*, several euphane-type triterpenoids were isolated and evaluated for their α-glucosidase inhibitory potential. Two of these compounds exhibited moderate activity, with IC_50_ values of 220 ± 11.1 μM and 86 ± 7.5 μM, compared to acarbose (IC_50_ = 370 ± 2.5 μM). Molecular docking confirmed that all triterpenoids could bind to the catalytic site of α-glucosidase, although with more flexible conformations than acarbose. The most active compounds displayed binding energies around −70 kcal/mol, corresponding to approximately 70% of the reference inhibitor’s activity. These findings represent the first report of α-glucosidase inhibition by euphane-type triterpenoids from *P. trimera* and suggest that side-chain oxygenation significantly contributes to inhibitory potency [98].

From the bark of *Diospyros melanoxylon*, enzyme-guided fractionation yielded lupenone, a pentacyclic triterpenoid with potent α-amylase inhibition. Kinetic analyses revealed a non-competitive mechanism (Ki = 30 μM), suggesting its potential to attenuate postprandial hyperglycemia [99].

Liquidambaric acid, a pentacyclic triterpenoid from *Liquidambar formosana*, exhibited strong α-glucosidase inhibition (IC_50_ = 0.12 mM) through a non-competitive mechanism. Docking studies indicated hydrogen bonding at an allosteric site, while 100 ns molecular dynamics simulations confirmed the stability of the enzyme–ligand complex. In a *Drosophila melanogaster* hyperglycemic model, liquidambaric acid significantly lowered glucose levels, showing good bioavailability and low toxicity [100].

Collectively, terpenoid-mediated inhibition of α-amylase and α-glucosidase is predominantly associated with non-competitive and allosteric mechanisms, reflecting their ability to bind outside the catalytic pocket and modulate enzyme activity through conformational changes. Their inhibitory effects are largely influenced by structural features such as oxidation patterns, side-chain functionalization, and the pentacyclic triterpene scaffold, which facilitate hydrogen bonding and hydrophobic interactions with enzyme residues. These interactions contribute to reduced catalytic efficiency and highlight terpenoids as promising scaffolds for the development of novel antidiabetic agents [95,96,97,98,99,100].

#### 4.1.4. Saponins

Saponins are a large and structurally diverse group of glycosides widely distributed in higher plants, though also found in certain marine organisms. They are characterized by their amphiphilic nature, consisting of a hydrophobic aglycone (sapogenin) linked to one or more hydrophilic sugar chains, which confers distinctive surface-active properties. Based on their aglycone backbone, saponins are generally classified into triterpenoid and steroidal types [101,102].

Saponins extracted from the stem bark of *Dialium guineense* showed significant in vitro inhibition of both α-amylase and α-glucosidase. Although tannin extracts were slightly more potent, saponin fractions still exhibited strong, concentration-dependent enzyme inhibition comparable to acarbose, supporting their potential as dual inhibitors of carbohydrate-hydrolyzing enzymes [103].

Two triterpenoid saponins isolated from the n-butanol fraction of *Polygonum capitatum* markedly inhibited α-amylase activity (51.9 ± 2.8% and 38.1 ± 2.2% inhibition). Molecular docking revealed strong binding affinities (−9.4 and −7.8 kcal/mol) at the enzyme’s active site, indicating that these saponins contribute to the traditional antidiabetic effects of *P. capitatum* [104].

Stigmastane-type steroidal saponins (vernogatiosides A–C) isolated from *Vernonia gratiosa* were identified as potent α-glucosidase inhibitors. Enzymatic and docking studies confirmed strong hydrogen bonding and hydrophobic interactions at the enzyme’s active site [105].

Triterpenoid saponins isolated from the root bark of *Aralia taibaiensis*, a traditional Chinese medicinal plant used for diabetes management, demonstrated potent dual inhibition of α-glucosidase and α-amylase. Among the thirteen isolated compounds, several showed exceptional inhibitory potency, with IC_50_ values as low as 0.72–0.93 μM against both enzymes, significantly surpassing acarbose. Structure–activity relationship analysis revealed that the number and position of sugar residues attached to the sapogenin core critically influenced inhibitory strength, underscoring the importance of glycosylation patterns in enzyme binding [106].

Triterpenoid saponins from *Chenopodium quinoa* husks showed strong α-glucosidase inhibition (IC_50_ = 32.62 mg/mL), about twice as potent as acarbose. Molecular docking indicated stable interactions (−12.7 to −7.7 kcal/mol) with key catalytic residues (His280, Asp307, Gly309, Asn247, Ser240). Glucuronic acid at C-3 and a carboxyl group at C-28 enhanced binding and induced conformational changes that reduced enzyme activity [107].

Saponins isolated from *Eleocharis dulcis* peels, stigmasterol glucoside, campesterol glucoside, and daucosterol, exhibited potent α-glucosidase inhibition (IC_50_ = 7.68, 10.03, and 5.67 mg L^−1^, respectively), far stronger than acarbose (91.50 mg L^−1^). Daucosterol, the most active compound, acted as a competitive inhibitor. Fluorescence spectroscopy confirmed complex formation with α-glucosidase, driven by hydrogen bonds and van der Waals forces (ΔG° = −29.92 kJ mol^−1^, ΔH° = −52.89 kJ mol^−1^, ΔS° = −76.65 J mol^−1^ K^−1^), demonstrating clear structure–activity relationships [108].

Overall, saponin-mediated inhibition of α-amylase and α-glucosidase is associated with both competitive and non-competitive mechanisms, depending on their structural features and binding mode. Their amphiphilic nature enables interaction with the enzyme active site as well as peripheral regions, facilitating hydrogen bonding, hydrophobic interactions, and van der Waals forces. The inhibitory potency of saponins is strongly influenced by the structure of the aglycone and the number, type, and position of sugar moieties, which modulate binding affinity, induce conformational changes, and ultimately affect enzyme activity and selectivity [104,105,106,107,108].

From a structure–activity relationship perspective, specific chemical features of plant secondary metabolites directly determine their mode of enzyme inhibition. The presence of hydroxyl groups enhances hydrogen bonding with catalytic residues, often favoring competitive or mixed-type inhibition. Increased molecular size and polymerization, as observed in tannins, limit access to the catalytic pocket and promote non-competitive or uncompetitive inhibition through allosteric binding or enzyme–substrate complex stabilization. Glycosylation influences solubility and binding orientation, modulating affinity toward either catalytic or peripheral sites. Similarly, oxidation patterns and functional group distribution in terpenoids and saponins affect their ability to induce conformational changes, often leading to allosteric inhibition [64,83,91].

These relationships demonstrate that enzyme inhibition is not only dependent on compound class but is primarily governed by precise structural determinants that dictate binding mode, interaction strength, and enzyme selectivity.

Table 1 and Table 2 present plant secondary metabolites that inhibit α-amylase and α-glucosidase, limited to compounds with reported IC_50_ values lower than 10 μM. The compounds are grouped by botanical source, enzymatic target, mechanism of action, and inhibitory activity determined in various experimental models, allowing comparison of their potential relevance in postprandial glucose control.

### 4.2. PTP1B Inhibitors

In contrast to carbohydrate-hydrolyzing enzymes, PTP1B inhibition primarily targets insulin signaling rather than glucose digestion. At the molecular level, plant secondary metabolites may inhibit PTP1B either by occupying the catalytic pocket, thereby preventing access of phosphotyrosine substrates, or by binding to adjacent or allosteric regions that alter the conformation and dynamics of catalytically important domains. Competitive inhibitors typically interact with residues within or near the active site, whereas non-competitive and mixed-type inhibitors may additionally affect loop mobility and substrate accommodation. Hydrogen bonding, π–π stacking, electrostatic interactions, and hydrophobic contacts are the main forces stabilizing PTP1B–ligand complexes and governing ligand binding and enzyme inhibition [110,111,112,113,114,115,116,117].

#### 4.2.1. Alkaloids

Alkaloids are a large and structurally diverse class of nitrogen-containing natural products found across multiple biological kingdoms, including plants, fungi, bacteria, and marine organisms. They are typically heterocyclic compounds biosynthesized from amino acid precursors and often contain primary, secondary, or tertiary amine groups [81].

Berberine, an isoquinoline alkaloid extensively used in traditional Chinese medicine for its antihyperglycemic effects, acts as a potent competitive inhibitor of PTP1B (Ki = 91.3 nM). Molecular docking revealed stable binding within the catalytic pocket, and in vitro assays showed 40% and 60% inhibition at 50 and 100 μM, respectively, supporting its insulin-mimetic and glucose-lowering activity [110,111].

Papaverine, a benzylisoquinoline alkaloid structurally related to berberine, also showed strong PTP1B inhibition (IC_50_ = 1.20 μM) and significantly reduced fasting blood glucose in Balb/c mice. Docking confirmed stable binding within the catalytic pocket, consistent with its potential as a natural antihyperglycemic agent [112].

Carbazole alkaloids from *Clausena anisum-olens* exhibited strong PTP1B inhibition (IC_50_ = 0.58–38.48 μM). The presence of a ketocarbonyl at C-1′ and a hydroxyl group at C-1 enhanced activity, while hydroxylation at C-2 reduced potency. Some compounds also inhibited α-glucosidase, indicating dual antidiabetic potential [113].

In *Tetradium ruticarpum*, two alkaloids, schinifoline and integrifoliodiol, were isolated and shown to inhibit both PTP1B and α-glucosidase. Schinifoline exhibited stronger activity (IC_50_ = 24.3 ± 0.8 μM for PTP1B and 92.1 ± 0.8 μM for α-glucosidase) than integrifoliodiol (IC_50_ = 47.7 ± 1.1 μM and 167.4 ± 0.4 μM, respectively). Density Functional Theory and molecular docking simulations revealed that hydrogen bonding with Arg254 and Arg676 residues played a key role in enzyme inhibition, inducing conformational changes that decreased catalytic efficiency [114].

Similarly, an in silico investigation on 25 indole alkaloids from *Rauvolfia serpentina* identified yohimbine as the most promising PTP1B inhibitor (ΔG = −5.03 kcal/mol; Ki = 206.06 mM), exhibiting binding affinity comparable to the reference compound ursolic acid (ΔG = −5.36 kcal/mol). Molecular docking revealed two π–π stacking interactions between the indole ring of yohimbine and the Tyr46 residue of PTP1B, which stabilized the ligand within the catalytic pocket. Twelve alkaloids showed the lowest binding energies and distinct interaction modes, suggesting R. serpentina as a potential natural source of indole-based PTP1B inhibitors with antidiabetic potential [118].

Novel indole alkaloids from *Evodia rutaecarpa* fruits displayed moderate inhibition of both α-glucosidase (IC_50_ = 23.9 μM) and PTP1B (IC_50_ = 75.8 μM) [119]. Two marine-derived meroterpenoid–alkaloid hybrids, Frondoplysin A and B, isolated from *Dysidea frondosa*, were potent PTP1B inhibitors (IC_50_ = 0.39 and 0.65 μM). Structural variation such as N-methylene substitution at C-19 reduced potency, underscoring the sensitivity of PTP1B–ligand interactions [120].

Overall, alkaloid-mediated inhibition of PTP1B is primarily associated with interactions at or near the catalytic pocket, frequently involving key residues responsible for substrate recognition. Isoquinoline and benzylisoquinoline alkaloids typically act as competitive inhibitors by occupying the active site, whereas indole and carbazole alkaloids may additionally engage in π–π stacking and hydrogen bonding, leading to conformational changes that reduce catalytic efficiency. Structural features such as heterocyclic frameworks, nitrogen-containing functional groups, and specific substitutions (hydroxyl or carbonyl groups) play a critical role in modulating binding affinity and inhibitory potency [110,111,112,113,114,120].

#### 4.2.2. Phenolics

Among the most abundant plant secondary metabolites are phenolic compounds, characterized by one or more hydroxylated aromatic rings. Their basic structural frameworks often derive from gallic acid, cinnamic acid, or catechin, which serve as precursors for a wide range of polyphenols, including flavonoids, lignans, and stilbenes [121].

Phloroglucinol derivatives from Syzygium cumini exhibited strong PTP1B inhibition (IC_50_ = 0.42–2.67 μM). Compounds with free hydroxyl groups at C-6 and C-8 and a C-2 side chain of 17 carbons were most active, while methylation at these positions reduced potency, confirming the importance of free hydroxyls [122]. Similarly, flavonoids isolated from *Psoralea corylifolia* also showed strong inhibitory activity [123].

Flavones from *Glycyrrhiza inflata* containing isoprenyl groups on the A and/or B rings were potent PTP1B inhibitors, with activity influenced by both hydroxyl position and prenyl substitution [124]. Guo et al. reported that licoflavone B and C (IC_50_ = 15.62 and 46.43 μM) were the most active among a series of flavonoids, where dual prenylation at C-6 (A ring) and C-3′ (B ring) enhanced inhibition [125].

Biflavonoids from Selaginella uncinata displayed strong inhibition (IC_50_ = 4.6 ± 0.5 μM). Activity increased with methyl substitution at C-6/C-6′ and 3–4″ or 3′–4″ interflavonoid linkages, while methoxy substitution or loss of the C2=C3 bond slightly reduced potency [126]. In contrast, a large flavonoid screening found generally weak inhibition (IC_50_ up to 184 μM), showing that hydroxylation at C-4′ only modestly increases activity [80].

Among lignans, eight new compounds from Viburnum cylindricum showed variable activity, with the S configuration at C-9 and oxidized substituents correlating with stronger inhibition [127]. Neolignans from *Eleutherococcus senticosus* containing a propenal moiety at C-4 exhibited the highest PTP1B inhibition, while relocation of this group to C-1 reduced potency [128].

Unsaturated alkynyl phenols from *Selaginella tamariscina* acted as non-competitive or mixed-type inhibitors (Ki < 15 μM). Methyl, methoxy, or hydroxymethyl substitution at C-15 enhanced activity relative to unsubstituted analogs [115].

Diarylheptanoid dimers from *Alpinia katsumada* also inhibited PTP1B, with activity dependent on the stereochemistry at C-3 and C-5. These compounds additionally inhibited α-glucosidase and glycogen phosphorylase a [129].

Phenolic acids trans-resveratrol and tricuspidatin B from *Polygonum cuspidatum* both inhibited PTP1B, the latter being more potent due to hydroxyl substitution at C-7/C-8 [130]. Stilbene dimers from *Cajanus cajan* showed activity dependent on hydroxyl–methoxy patterns and keto substitution at C-2′, while isoprenylation reduced potency [131].

Stilbene glucosides from *Polygonum multiflorum* (IC_50_ = 1.2–4.6 μM) and prenylated stilbenes from *Artocarpus styracifolius* also displayed strong inhibition, influenced by hydrogen atom orientation at H-6/H-13 PTP1B [132]. Curcusinol, a stilbene oligomer from *Clematis hexapetala*, inhibited PTP1B (IC_50_ = 4.62 μM) and suppressed TNF-α secretion (IC_50_ = 3.42 μM), indicating dual antidiabetic and anti-inflammatory potential [133].

From the marine-derived fungus *Aspergillus puniceus*, four xanthones and five anthraquinones showed strong PTP1B inhibition. In xanthones, methoxy substitution at C-1 reduced activity, while in anthraquinones, potency depended on the type and length of the side chain at C-2 [134]. Similarly, xanthones from *Cratoxylum cochinchinense* exhibited dual PTP1B and α-glucosidase inhibition; the presence of a geranyl group at C-10 enhanced potency, while isoprenyl loss diminished activity. These acted as competitive PTP1B and mixed-type α-glucosidase inhibitors [109].

Taken together, phenolic compounds represent a structurally and functionally diverse group of natural PTP1B inhibitors, encompassing subclasses such as flavonoids, lignans, stilbenes, xanthones, and related derivatives. Their inhibitory activity is strongly influenced by the number and position of hydroxyl groups, the degree of prenylation or methoxylation, and the presence of conjugated double bonds, which collectively influence binding affinity and enzyme selectivity. These findings highlight phenolics as versatile scaffolds for developing multitarget antidiabetic agents [115,122,124,126,127,130,134].

#### 4.2.3. Chalcones

Following the diverse phenolic inhibitors, chalcones emerge as structurally related compounds distinguished by their open-chain flavonoid framework and notable PTP1B inhibitory potential. Chalcones are flavonoid-type phytochemicals, often referred to as “open-chain flavonoids,” and are biosynthesized via the shikimate pathway. They are regarded as the biosynthetic precursors of flavonoids and are typically characterized by an α, β-unsaturated carbonyl system linking two aromatic rings. Naturally occurring chalcones frequently contain one or more phenolic hydroxyl groups, as well as prenyl or geranyl substitutions on the aromatic rings, structural features that contribute to their wide range of biological and therapeutic activities [135].

Several natural chalcones have been identified as potent PTP1B inhibitors.

Five chalcones isolated from *Alpinia katsumadai* exhibited significant inhibitory activity, with IC_50_ values ranging from 22.5 to 89.2 μM. Structural analysis indicated that a hydroxyl group at the C-3 position enhances enzyme inhibition, while chiral configuration influences binding affinity. These compounds also produced hypoglycemic effects in db/db mice, supporting their potential as natural antidiabetic agents [136].

Bioassay-guided fractionation of the chloroform extract of *Morus bombycis* yielded three chalcone-derived Diels–Alder adducts, kuwanons J, R, and V, which showed strong PTP1B inhibition (IC_50_ = 4.3–13.8 μM). The inhibitory potency followed the order J > R > V, indicating that increased hydroxylation enhances activity. Kinetic analysis revealed a mixed-type inhibition mechanism, suggesting interaction with both catalytic and allosteric sites of PTP1B [116].

Chalcone–monoterpene hybrids isolated from the buds of *Cleistocalyx operculatus* also showed potent PTP1B inhibition (IC_50_ = 0.9–3.9 μM). Kinetic and docking analyses revealed a mixed-type inhibition mechanism, suggesting interaction with both catalytic and allosteric sites of the enzyme [117].

Broussochalcone, isolated from *Broussonetia papyrifera*, exhibited potent PTP1B inhibition (IC_50_ = 21.5 μM). The presence of two hydroxyl groups on each aromatic ring was identified as a key determinant of activity, confirming that inhibitory potency increases with the number of hydroxyl substituents.

Licochalcone A, isolated from *Glycyrrhiza inflata*, along with its semi-synthetic derivatives, also showed significant PTP1B inhibition. The methylated derivative displayed the highest potency, suggesting that methyl substitution enhances enzyme binding and inhibitory strength [125,137].

Two chalcones isolated from *Macaranga denticulata*, a dimeric macdentichalcone and its proposed monomeric precursor, 1-(5,7-dihydroxy-2,2,6-trimethyl-2H-1-benzopyran-8-yl)-3-phenyl-2-propen-1-one, exhibited comparable PTP1B inhibition (IC_50_ = 21–22 μM), indicating that dimerization does not significantly affect inhibitory activity [138].

Overall, chalcones represent an important subclass of flavonoid-type compounds with strong PTP1B inhibitory potential. Structural variations such as hydroxylation, methylation, and hybridization with terpenoid moieties significantly influence enzyme binding, supporting their value as lead scaffolds for the development of novel antidiabetic agents [116,117,137,138].

Collectively, these findings indicate that the inhibitory activity of plant secondary metabolites against PTP1B is strongly influenced by SAR. Across different phytochemical classes, specific structural features play a critical role in determining binding affinity, inhibition mechanism, and enzyme selectivity. In alkaloids, heterocyclic frameworks, the presence of nitrogen atoms, and functional groups such as hydroxyl or carbonyl moieties influence interactions with catalytic residues, often associated with competitive inhibition. In phenolic compounds, the number and position of hydroxyl groups, degree of prenylation or methoxylation, and the presence of conjugated aromatic systems significantly enhance hydrogen bonding, π–π stacking, and electrostatic interactions, thereby modulating inhibitory potency. Chalcones exhibit distinct SAR characteristics driven by their α,β-unsaturated carbonyl system and hydroxyl substitution pattern, which facilitate both catalytic and allosteric binding, often resulting in mixed-type inhibition. Overall, these structural determinants strongly influence ligand–enzyme interactions, binding mode, and inhibitory efficiency, providing a mechanistic basis for the rational design of novel PTP1B inhibitors with improved antidiabetic potential [110,111,112,113,114,117,118,119,120,122,123,126,136].

Table 3 summarizes plant-derived secondary metabolites reported to inhibit PTP1B, limited to compounds with reported IC_50_ values lower than 10 μM. The listed compounds are organized according to their botanical source, mechanism of action, and reported inhibitory activity in different experimental models, highlighting natural inhibitors with potential to improve insulin sensitivity and glucose homeostasis.

### 4.3. DPP-4 Inhibitors

Plant-derived DPP-4 inhibitors generally act by interacting with the substrate-binding cavity of the enzyme, thereby limiting access of incretin substrates such as GLP-1 and GIP to the catalytic region. In most cases, this leads to competitive inhibition when phytochemicals bind within the active-site region, although mixed-type or non-competitive inhibition may also occur when binding involves adjacent regions that influence substrate recognition or catalytic turnover. Structurally, DPP-4 contains a catalytic triad (Ser630, Asp708, and His740) together with several substrate-recognition subsites, mainly S1, S2, and S3, which shape ligand affinity and inhibitory behavior. At the molecular level, these interactions are typically stabilized by hydrogen bonding, hydrophobic contacts, π–π stacking, and electrostatic interactions with residues located in or near the binding cavity, including Glu205, Glu206, Tyr547, Phe357, Ser630, Tyr662, and Tyr666. Overall, the inhibitory profile of phytochemicals against DPP-4 depends not only on binding affinity, but also on structural features such as scaffold geometry, degree of substitution, and conformational flexibility [140,141,142].

#### 4.3.1. Flavonoids

Evaluations with purified enzyme show that flavonoids produce measurable inhibition of DPP-4, and docking simulations frequently support binding geometries compatible with active-site occupation [143].

Reported potencies vary widely depending on scaffold and assay conditions. Among the more active representatives, naringenin, luteolin, and apigenin showed submicromolar inhibition, whereas glycosylated derivatives such as naringin and cyanidin-3-O-glucoside were less potent, suggesting that glycosylation generally attenuates activity [144,145,146].

Flavonols showed more heterogeneous effects: quercetin and galangin inhibited DPP-4 with IC_50_ = 4.02 µM and 40.13 µM, whereas isoquercitrin was less potent (IC_50_ = 96.8 µM) [147,148,149]. Myricetin showed micromolar potency in a purified-enzyme system (IC_50_ = 4.8 µM), with reduced apparent activity in protein-rich matrices, suggesting matrix-binding effects [150]. Additional flavonoids, including rutin and related glycosides, generally showed only moderate inhibition, and favorable docking scores were not always matched by equivalent in vitro activity [151,152,153,154,155,156,157].

Kinetic and interaction studies further clarified how flavonoids inhibit DPP-4. Myricetin acted as a reversible non-competitive inhibitor, whereas hyperoside, narcissoside, cyanidin 3-O-glucoside, and isoliquiritigenin showed mixed-type inhibition. Docking and fluorescence-quenching analyses supported these results, showing different binding locations for myricetin compared with the other flavonoids. These findings indicate that flavonoids can inhibit DPP-4 through distinct kinetic mechanisms depending on their binding mode [140].

Consistent with their suitability as DPP-4 ligands, catechin isolated from *Withania somnifera* displayed a predicted binding energy of −6.60 kcal/mol, forming 13 hydrogen bonds to residues within the binding region (e.g., Glu347, Met348, Ser349, Thr351, Ile375, Asn377, Glu378, Gly380, Asp588) [158,159]. Independent docking using a crystallographic DPP-4 structure ranked catechin and epicatechin among higher-scoring phytochemicals (predicted ΔG = −8.20 and −8.01 kcal·mol^−1^; estimated Ki = 0.97 and 1.36 µM), with multiple hydrogen bonds (e.g., Tyr195/Tyr211, Asn170, Glu191/Glu204) and hydrophobic contacts consistent with stable complexes [160].

Green-tea catechins align with these trends. EGCG was predicted to engage the active region and remain stable over a 100 ns molecular-dynamics trajectory, and it inhibited DPP-4 with an IC_50_ of 28 µM. Other catechins were less potent (ECG = 106.8 µM; EC = 280.8 µM; (+)-catechin = 381.3 µM; EGC = 567.5 µM), while the benchmark gliptin sitagliptin acted in the nanomolar range (IC_50_ = 1.393 nM) [161].

Beyond direct enzyme inhibition, EGCG also reduced DPP4 expression and inflammatory signaling in cellular models, indicating that its metabolic effects may extend beyond direct catalytic blockade [162,163].

Overall, the aggregate evidence positions selected flavonoid chemotypes as capable DPP-4 modulators reaching micromolar inhibition, whereas many glycosylated congeners (including several catechins) display modest to weak potency. For catechins specifically, convergent docking/MD support active-site compatibility, yet in-enzyme potency is typically modest relative to reference gliptins.

#### 4.3.2. Lignans

Lignans are plant polyphenols formed by oxidative coupling of two C6–C3 phenylpropanoid units, and their dimeric scaffold confers marked antioxidant properties and broad biological activity [164].

In vitro studies indicate that several lignans can inhibit DPP-4, generally with moderate efficacy, while showing limited effects on other carbohydrate-hydrolyzing enzymes [165].

Among the investigated compounds, matairesinol stands out due to available in vivo evidence. In streptozotocin-induced diabetic rats, it improved glycemic control and insulin levels, suggesting a potential metabolic benefit. Docking analyses further supported its interaction with the DPP-4 catalytic site, consistent with possible target engagement [166].

However, DPP-4 inhibition is not a consistent feature across lignans. In other studies, compounds such as schisandrin C, anwuligan, and magnolol showed negligible activity (IC_50_ > 100 µM), indicating minimal interaction with the enzyme under comparable conditions [167].

Collectively, available data suggest a context-dependent modulation of DPP-4 by certain lignans and support the need for standardized assays and in vivo target validation to determine which structural motifs effectively translate biochemical activity into glycemic benefit.

#### 4.3.3. Stilbenes and Derivatives

Stilbenes are plant-derived phenolic compounds widely distributed in grapes as well as in a variety of other edible and medicinal plants [168]. They share a characteristic 1,2-diphenylethylene core and occur either as simple monomeric molecules or as more complex oligomeric structures, conventionally classified into mono-, di-, tri-, tetra-, polymeric, and heteromeric stilbenes [169,170]. These molecules adopt both cis and trans configurations, with the trans isomer representing the dominant and more stable form in nature [171]. Through this structural diversity, stilbenes contribute significantly to the phytochemical profile and potential bioactivity of stilbene-rich plant sources.

Resveratrol is one of the most extensively studied stilbenes and has been reported as a DPP-4 inhibitor, although its potency appears to vary considerably depending on the assay system. While some studies describe strong inhibition in enzymatic assays, others report more moderate activity. In vivo, resveratrol has been shown to reduce circulating DPP-4 levels and improve glycemic parameters in diabetic models, suggesting a functional link between enzyme modulation and metabolic effects [144,172].

Oligomeric stilbenes also exhibit DPP-4 inhibitory activity. Among these, (−)-vitisin B showed moderate inhibition in vitro and demonstrated beneficial metabolic effects in diabetic animal models, including improved glycemic control and insulin response. However, its potency remains substantially lower than that of synthetic gliptins [173,174].

Structure–activity relationship studies further indicate that DPP-4 inhibition can be enhanced by structural modifications such as geranyl substitution, whereas parent stilbenes generally show only modest activity [175]. Additional derivatives have shown measurable inhibition only at high concentrations, raising questions about their physiological relevance [176].

Similar discrepancies are observed for piceatannol, which exhibited weak enzymatic inhibition despite favorable docking predictions, highlighting the limitations of in silico approaches in predicting biological activity [175,176,177,178].

Taken together, these findings indicate that stilbenes, including resveratrol as well as selected oligomeric and geranylated derivatives, exert modulatory effects on DPP-4 activity and thereby improve glycemic parameters in experimental models.

#### 4.3.4. Terpenoids

As one of the largest and most structurally diverse classes of natural products, terpenoids are increasingly assessed for DPP-4 inhibitory potential [179,180].

Recent in silico investigations have identified several natural terpenoids as promising DPP-4 inhibitors, displaying favorable binding affinities, stable ligand–enzyme interaction profiles, and acceptable pharmacokinetic properties, thereby supporting their potential development as lead molecules for T2DM therapy [181].

Large-scale virtual screening efforts have identified several candidates, including asiatic acid and aucubin, as potential DPP-4 binders, and similar trends have been observed across independent datasets [182,183,184,185,186].

In *Urena lobata*, terpenoid phytosterols were also implicated in DPP-4 inhibition. Docking analyses predicted favorable interactions of these sterols with DPP-4, with binding free energies of approximately −7.42 kcal/mol for stigmasterol (estimated Ki = 3.62 mM) and −6.59 kcal/mol for β-sitosterol (estimated Ki = 14.67 mM), supporting their contribution to the observed activity [187].

From a translational perspective, in vitro evaluation of terpenoid constituents from two medicinal plants identified multiple triterpenes with measurable DPP-4 inhibition.

From *Fagonia cretica*, quinovic acid (IC_50_ = 30.7 µM) and its glycosides—quinovic acid-3β-O-β-D-glucopyranosyl-(28→1)-β-D-glucopyranosyl ester (IC_50_ = 23.5 µM) and quinovic acid-3β-O-β-D-glycopyranoside (IC_50_ = 57.9 µM). In contrast, the sterol stigmasterol showed only weak inhibition (IC_50_ > 100 µM). From *Hedera nepalensis*, lupeol (IC_50_ = 31.6 µM) was active [187]. Likewise, stellasterol displayed poor enzymatic inhibition despite predicted active-site binding, underscoring the discrepancy between computational and biochemical results [188].

Overall, the profile of terpenoids as DPP-4 inhibitors remains promising, but moving from docking affinities and moderate in vitro activities to real therapeutic candidates will depend on targeted structural optimization and in vivo functional confirmation.

#### 4.3.5. Alkaloids

Alkaloids display wide-ranging pharmacology and serve as chemically privileged templates in modern drug discovery [189]. 

Berberine is one of the most consistently reported alkaloids with DPP-4 inhibitory activity. Enzymatic studies indicate low-micromolar potency, supporting direct enzyme inhibition, while computational analyses suggest stable binding within the DPP-4 active site. Related isoquinoline alkaloids further support the relevance of this chemotype for DPP-4 targeting [190,191,192,193,194].

The alkaloid lobeline demonstrated consistent activity across experimental models, including substantial inhibition of plasma DPP-4 activity in diabetic mice, confirming target engagement under physiological conditions [195].

Similarly, the indole alkaloid 16,17-dihydro-17β-hydroxy-isomitraphylline showed dose-dependent enzyme inhibition in vitro and increased circulating active GLP-1 levels in vivo [196].

In contrast, several other alkaloids, including simple ephedrine derivatives, exhibited only weak to moderate inhibition, often in the high micromolar to millimolar range [197].

Numerous additional compounds have been proposed as DPP-4 inhibitors based on docking studies; however, these findings generally lack experimental validation [198,199].

Finally, in a study on *Castanospermum australe* seeds, the ethanolic extract demonstrated DPP-4 inhibition. Three alkaloids were identified and evaluated by molecular docking against DPP-4, castanospermine, 7-deoxy-6-epi-castanospermine, and australine, with 7-deoxy-6-epi-castanospermine exhibiting the most favorable binding features and docking performance comparable to berberine [200].

In summary, the integration of biochemical and computational results points to alkaloids, especially berberine, as compelling lead frameworks for DPP-4 inhibitors.

Compared with other diabetes-related targets such as α-amylase, α-glucosidase, and PTP1B, plant-derived DPP-4 inhibitors remain less well characterized kinetically. Their mechanisms are therefore interpreted mainly from combined evidence provided by enzyme assays, docking, fluorescence-based binding studies, and molecular dynamics simulations.

Despite these limitations, the available data suggest that DPP-4 inhibition by plant secondary metabolites is strongly structure-dependent. In flavonoids, selected hydroxylation patterns and preservation of the conjugated C2=C3–4-carbonyl system generally favor activity, whereas methylation often reduces it and glycosylation shows variable effects depending on scaffold and substitution position. In stilbenes, terpenoids, and alkaloids, emerging evidence suggests that the balance between polar substituents and the lipophilic scaffold may influence binding affinity and inhibitory potency [140,141,175,201].

For DPP 4, no separate table was compiled because, unlike for α amylase, α glucosidase, and PTP1B, only a single plant-derived secondary metabolite with an IC_50_ below 10 µM was identified: emodin an anthraquinone, from *Rheum palmatum*, with an IC_50_ of 5.76 µM without detectable effects on DPP-8/9 [202]. While other compounds with IC_50_ < 10 µM have been reported, these were tested as pure chemical standards rather than being isolated from plants. Most plant extracts and phytochemicals exhibited weak to moderate inhibitory activity, with IC_50_ values predominantly above the low micromolar range. Therefore, a potency-based table analogous to those for the other enzymes was not feasible, and notable findings and structure–activity trends are discussed in the main text.

## 5. Critical Considerations and Limitations

Despite the growing body of evidence supporting the antidiabetic potential of plant secondary metabolites, several limitations should be considered when interpreting these findings. First, considerable variability in IC_50_ values is often reported across studies, even for the same compound [64,70,76,79]. This is largely due to differences in enzyme source, substrate type, assay conditions, and experimental protocols, which makes direct comparison of inhibitory potency difficult. Second, most of the available data are derived from in vitro enzyme assays or in silico studies, while in vivo activity may differ substantially. Factors such as bioavailability, intestinal absorption, metabolism, plasma protein binding, and chemical stability can significantly influence biological effects [79,155,182]. As a result, compounds showing strong inhibition in isolated enzyme systems do not always exhibit comparable efficacy under physiological conditions.

Reproducibility also remains an important issue, particularly for docking-based studies, where results may vary depending on the protein model, scoring function, and computational parameters used, and where independent validation is often limited. In addition, many plant-derived metabolites present unfavorable pharmacokinetic properties, including low solubility, rapid biotransformation, and limited systemic exposure, which restrict their translational potential [203,204]. Various strategies, such as nanoformulations, encapsulation approaches, prodrug design, or structural modification, have been proposed to improve stability and absorption.

Finally, although the available findings are promising, their therapeutic relevance should be interpreted with caution. Most of the current evidence comes from preclinical studies or isolated enzyme models, while robust clinical data remain scarce. Therefore, future research should focus on standardized methodologies, integrated in vitro–in vivo approaches, detailed pharmacokinetic characterization, and well-designed clinical studies in order to better define the real antidiabetic potential of plant secondary metabolites.

## 6. Conclusions

This review consolidates current knowledge on plant-derived compounds and extracts with antidiabetic potential, focusing on their effects on enzymes involved in glucose metabolism/regulation. Flavonoids, tannins, terpenoids, alkaloids, and phenolics show strong and structure-dependent inhibition of α-amylase, α-glucosidase, and PTP1B, demonstrating how natural products can modulate enzymatic targets similarly or complementarily to conventional antidiabetic drugs. Potent plant-derived DPP-4 inhibitors are rare, with most compounds showing only modest activity, highlighting a gap in the discovery of natural modulators for this enzyme. Overall, these findings emphasize the multifunctional potential of plant secondary metabolites as multi-target antidiabetic agents and support further research to optimize their structures, explore additional molecular and cellular targets, and validate their effects in vivo.

## Figures and Tables

**Table 1:** Secondary metabolites from plant sources reported as α-amylase potent inhibitors (IC_50_ < 10 μM).

Plant Species (Latin Name)	Secondary Metabolite(s)	Target Enzyme	Inhibition Mechanism	Reported Activity IC50 (μM)	Experimental Model	Ref.
*Sambucus nigra*	Quercetin	Porcine pancreatic α-amylase	N/A	2.1 ± 0.5	In vitro enzyme assay	[76]
	Kaempferol			3.6 ± 1.1		
	Rutin			4.1 ± 0.8		
*Camellia sinensis*	Kaempferol diglycoside	Porcine pancreatic α-amylase	Hydrogen bonding and van der Waals interactions	0.09 ± 0.02	In vitro enzyme assay; Molecular docking; Intrinsic fluorescence quenching	[78]

IC50—inhibitory concentration 50; N/A—not applicable/information not reported.

**Table 2:** Secondary metabolites from plant sources reported as α-glucosidase potent inhibitors (IC_50_ < 10 μM).

Plant Species (Latin Name)	Secondary Metabolite(s)	Target Enzyme	Inhibition Mechanism	Reported Activity IC50 (μM)	Experimental Model	Ref.
*Hovenia dulcis*	Myricetin	α-glucosidase	Reversible, non-competitive	9.43	In vitro enzyme assay	[70]
*Sambucus nigra*	Quercetin	*Saccharomyces cerevisiae* α-glucosidase	N/A	2.6 ± 0.9	In vitro enzyme assay	[76]
	Kaempferol			4.6 ± 2.3		
	Rutin			4.5 ± 1.2		
*Astragalus membranaceus*	Quercetin	*Saccharomyces cerevisiae* α-glucosidase	Mixed-type inhibition; Hydrogen bonding and π–π stacking	6.65 ± 0.43	In vitro enzyme assay; Molecular docking	[64]
*Potentilla speciosa var. speciosa*	Cinnamtannin D1	α-glucosidase	Non-competitive	0.849 ± 0.014	In vitro enzyme assay; Molecular modeling studies	[82]
*Geranium asphodeloides*	1,2,4-tri-O-galloyl-β-d-glucopyranose	α-glucosidase	Uncompetitive	0.95 ± 0.07	In vitro enzyme assay; Molecular modeling studies	[83]
*Melastoma dodecandrum*	Casuarictin	*Saccharomyces cerevisiae* α-glucosidase	Competitive inhibition; Hydrogen bonding	0.224	In vitro enzyme assay; Fluorescence quenching analysis	[85]
*Eleocharis dulcis*	Daucosterol	*Saccharomyces cerevisiae* α-glucosidase	Competitive inhibition Hydrogen bonds and van der Waals forces interactions	9.83	In vitro enzyme assay; fluorescence quenching	[108]
*Cratoxylum cochinchinense*	cratoxanthone A	α-glucosidase	Mixed-type inhibition	4.8	In vitro enzyme assay	[109]
	α-mangostin			5.7		
	γ-mangostin			1.7		

IC50—inhibitory concentration 50; N/A—not applicable/information not reported.

**Table 3:** Secondary metabolites from plant sources reported as PTP1B potent inhibitors (IC_50_ < 10 μM).

Plant Species (Latin Name)	Secondary Metabolite(s)	Inhibition Mechanism	Reported Activity IC50 (μM)	Experimental Model	Ref.
*Clausena anisum-olens*	Clausenanisine A	N/A	0.58 ± 0.05	In vitro enzyme assay	[113]
	Clausenanisine B		0.87 ± 0.06		
	Euchrestifoline		1.28 ± 0.07		
*Dysidea frondosa*	Frondoplysin A	N/A	0.39	In vitro enzyme assay	[120]
	Frondoplysin B		0.65		
*Glycyrrhiza inflata*	Licoagrochalcone A	N/A	0.97	In vitro enzyme assay	[124]
	kanzonol C		0.45		
	2′-hydroxyisolupalbigenin		0.5		
	gancaonin Q		0.55		
	glisoflavanone		0.84		
	glabrol		0.31		
	licoflavone C		46.43		
*Selaginella uncinata*	(2*S*) 2,3-dihydro-5,5″,7,7″,4′-pentahydroxy-6,6″-dimethyl-[3′-O-4‴]-biflavone	Non-competitive inhibition	4.6 ± 0.5	In vitro enzyme assay; Molecular docking	[139]
	(2″*S*) chrysocauloflavone		5.5 ± 0.7		
	delicaflavone		6.2 ± 0.5		
*Viburnum cylindricum*	Viburindrin D	N/A	6.14 ± 0.21	In vitro enzyme assay	[127]
	Viburindrin G		7.73 ± 0.15		
*Selaginella tamariscina*	Selariscinin A	N/A	4.8	In vitro enzyme assay	[115]
*Polygonum cuspidatum*	Emodin	Noncompetitive	7.6 ± 0.1	In vitro enzyme assay	[130]
	Tricuspidatin B	Mixed-competitive	6.3 ± 0.2		
*Artocarpus styracifolius*	Tyrastilbene A	Mixed-competitive	4.52	In vitro enzyme assay; Molecular docking	[132]
	Styrastilbene B		2.42		
	Hypargystilbene B		8.80		
	Trans-oxyresveratro		8.43		
*Clematis hexapetala*	Curcusinol	N/A	4.62	In vitro enzyme assay	[133]
*Aspergillus puniceus*	7-Chloro versicolorin A	N/A	8.0	In vitro enzyme assay	[134]
	Austocystins H		0.9		
	Austocystins B		1.8		
	Austocystins D		1.7		
	Averufin		1.1		
	Methyl-averantin		0.6		
	Averufanin		5.9		
*Cratoxylum cochinchinense*	Caratoxanthone A	Competitive inhibition	2.4	In vitro enzyme assay	[109]
	Cochinechinone A		5.2		
	α-Mangostin		5.5		
	γ-Mangostin		2.8		
	Pruniflorone S		7.05		
*Morus bombycis*	Albafuran A	Mixed-competitive	9.2 ± 0.7	In vitro enzyme assay	[116]
	Mulberrofuran W		2.7 ± 0.3		
	Kuwanon J		2.7 ± 0.6		
	Kuwanon R		8.2 ± 0.9		
	Mulberrofuran D		4.3 ± 0.5		

IC50—inhibitory concentration 50; N/A—not applicable/information not reported.

## Data Availability

All data generated or analyzed during this study are included in this published article.
